# Quality of life assessments in clinical practice using either the EORTC-QLQ-C30 or the SEIOQL-DW: a randomized study

**DOI:** 10.1186/s41687-021-00315-z

**Published:** 2021-07-14

**Authors:** Åsa Kettis, Hanna Fagerlind, Jan-Erik Frödin, Bengt Glimelius, Lena Ring

**Affiliations:** 1 Division for Quality Enhancement, Uppsala University, Uppsala, Sweden; 2 Primary Care and Health, Uppsala County Council, Stockholm, Sweden; 3 Department of Oncology and Pathology, Karolinska Institutet, Stockholm, Sweden; 4 Department of Immunology, Genetics and Pathology, Uppsala University, Uppsala, Sweden; 5 Healthcare Sciences and e-Health, Department of Women’s and Children’s Health, Uppsala University, Uppsala, Sweden

**Keywords:** Oncology, Clinical practice, EORTC-QLQ-C30, SEIQOL-DW, Individualized quality of life

## Abstract

**Background:**

Effective patient-physician communication can improve patient understanding, agreement on treatment and adherence. This may, in turn, impact on clinical outcomes and patient quality of life (QoL). One way to improve communication is by using patient-reported outcome measures (PROMs). Heretofore, studies of the impact of using PROMs in clinical practice have mostly evaluated the use of standardized PROMs. However, there is reason to believe that individualized instruments may be more appropriate for this purpose.

The aim of this study is to compare the effectiveness of the standardized QoL-instrument, the *European Organization for Research and Treatment of Cancer Quality of Life C-30* (EORTC-QOL-C30) and the individualized QoL instrument, the *Schedule for the Evaluation of Individual Quality of Life-Direct Weighting* (SEIQoL-DW), in clinical practice.

**Methods:**

In a prospective, open-label, controlled intervention study at two hospital out-patient clinics, 390 patients with gastrointestinal cancer were randomly assigned either to complete the EORTC-QOL-C30 or the SEIQoL-DW immediately before the consultation, with their responses being shared with their physician. This was repeated in 3–5 consultations over a period of 4–6 months. The primary outcome measure was patients’ health-related QoL, as measured by FACIT-G. Patients’ satisfaction with the consultation and survival were secondary outcomes.

**Results:**

There was no significant difference between the groups with regard to study outcomes. Neither intervention instrument resulted in any significant changes in health-related QoL, or in any of the secondary outcomes, over time. This may reflect either a genuine lack of effect or sub-optimization of the intervention. Since there was no comparison to standard care an effect in terms of lack of deterioration over time cannot be excluded.

**Conclusions:**

Future studies should focus on the implementation process, including the training of physicians to use the instruments and their motivation for doing so. The effects of situational use of standardized or individualized instruments should also be explored. The effectiveness of the different approaches may depend on contextual factors including physician and patient preferences.

## Background

Patient-physician communication is important and complex – “a curious and ubiquitous component of a cancer care system” [1] – and may have an impact on clinical outcomes [2]. Effective communication can improve patient understanding, physician–patient agreement on treatment, and adherence to treatment. This may, in turn, impact on clinical outcomes and patient quality of life (QoL). For example, a patient communication skills intervention might stimulate patients to talk about pain, which might, in turn, prompt the physician to change pain medication, resulting in better pain control and improved QoL.

Poor communication might, on the other hand, mean that important information goes undetected, and patients feel neglected. Cancer patients describing their physicians as disengaged, hurried or lacking in empathy are more likely to report hopelessness and emotional distress; they find that only the medical aspects of the disease receives attention, not their emotional concerns [3]. Poor communication may also undermine shared decision making in sensitive treatment decisions [4]. Systematic observations of patient-physician consultations confirm that chronic problems and psychosocial aspects of illness tend to receive less attention than acute medical problems [5], although both patients and physicians agree on their importance [6].

There are different means of improving patient-physician communication. Communication skills training significantly improves physician skills and increases patient satisfaction with the physician’s performance [7]. It is also possible to systematically monitor patients with different patient-reported outcome measures (PROMs). According to a comprehensive conceptual framework proposed by Santana et al. [8] there are a number of potential benefits associated with the use of PROMs in chronic care management. These relate to communication, shared clinical decision making, patient management and patient outcomes [8]. Other potential benefits include increased patient activation and improved clinician and patient satisfaction, as well as improved treatment adherence. Empirical studies confirm these benefits, although the impact on health outcomes is inconclusive [9–11].

A number of studies have used *standardized* QoL instruments, such as EORTC-QOL-C30 and FACT-G for the intervention [12–14]. However, standardized QoL instruments may not be valid since they capture health status rather than QoL as perceived by the patient [15]. Another shortcoming is that these instruments assume that the weightings of different components of QoL are the same for all individuals. This is contradicted by findings showing that the definition of QoL is highly individual. Patients vary in the weights that they attach to different aspects of life. Furthermore such weights may change within patients over time [16].

The use of a standardized PROMs may support patients who find it hard to introduce sensitive issues spontaneously [17]. On the other hand, some physicians find that standardized PROMs constrain the patient-physician relationship by not taking account of the complex nature of patients’ problems. Sometimes physicians even adjust PROMs in order to make them more fit for purpose, thereby undermining their standardization. This is especially common if the use of a specific PROM is mandated or incentivized [17]. Given this, *individualized* PROMs might be preferable for monitoring QoL in clinical practice [18]. Individualized PROMs encourage patients to share information that really matters to them [17], i.e. they are intentionally non-standardized and allow for self-nomination of areas of importance to the individual. These types of instrument are very different from standardized instruments. For individualized instruments the domains are generated by patients who are asked to nominate and weight the most important elements of their QoL, such as “family”, “hobbies”, “profession/ occupation”, “social life”, etc. The use of individualized QoL assessments [19] might facilitate better communication and problem identification than standardized instruments, since they adopt a more patient-centered approach [20].

The individualized instrument SEIQoL-DW (The Schedule for the Evaluation of Individual Quality of Life) is an acceptable measure of QoL in different patient populations and among caregivers [21–24]. For example, patients with ALS and HIV/AIDS perceive the validity of SEIQoL-DW in measuring their QoL to be higher than that of traditional health-related QoL-instruments such as the SIP (Sickness Impact Profile) and SF-36 (Short Form-36) [22, 23]. The ability of the instrument to capture the individual’s definition of their own QoL is especially important in cancer care, since changes in state of health might modify the individual’s internal standards, values and even their conceptualization of QoL [25]. It has been shown that individualized measures capture more domains i.e., have a greater heterogeneity in QOL contributors which is not reflected in standard QOL measures [26]. Furthermore, individualized instrument have been shown to be acceptable for use in cancer populations with regard to their fit-for-purpose properties [27].

The SEIQoL-DW has been tested for use in clinical practice in a qualitative study. Gastro-intestinal cancer patients were asked to fill in the SEIQoL-DW directly before visiting their oncologist [28]. A touch-screen computer version of the instrument was used. This had been tested for feasibility in cancer patients and shown to provide the same results as a paper and pen version [29]. Upon completion of the instrument, the results were printed and made available to both the physician and the patient during the consultation. When interviewed afterwards, the oncologists were cautiously positive about the potential of routine use of SEIQoL-DW in clinical practice. They believed that it might facilitate detection of patients’ areas of concern and thereby support the monitoring of the patients’ QOL. Patients also mentioned these benefits, but also reported that the instrument encouraged them to reflect upon their own overall life situation. In addition, they felt that it contributed to them being seen as a “whole person” by the physician and to empowering them by making it easier for them to voice their concerns. However, the QoL results were only brought up in a few consultations, mainly due to time pressure and the physician not always understanding how to use them [28].

As outlined above, relatively few studies have shown that the routine use of PROMs improves HRQL. Our hypothesis was that an individualized instrument might contribute to a more effective intervention with regard to having an impact on QoL outcomes. This would be mediated by improved communication, based on the feed-back of HRQL data, leading to better treatment and care and QoL. Thus, there is reason to hypothesize that an individualized approach using the SEIQoL-DW would be more effective than a standardized approach using EORTC-QOL-C30 in improving communication and QoL, given that increased attention might be paid to interpretation and implementation.

## Aims

The study aimed to compare the effectiveness of the EORTC-QOL-C30 and the individualized instrument the SEIQoL-DW as means of individualizing and improving cancer care in clinical practice. The primary outcome was patients’ health-related quality of life (HRQoL), while secondary outcomes were survival and patients’ satisfaction with patient-physician consultation and communication.

## Methods

### Population

Eligible patients were over 18 years, had a gastrointestinal (GI) cancer and were scheduled to regularly visit the outpatient units at the Departments of Oncology in Uppsala (Uppsala University Hospital) or in Stockholm (Karolinska University Hospital) during the coming four to 6 months. They could either receive neo-adjuvant therapy prior to surgery, adjuvant chemotherapy after primary surgery, palliative chemotherapy for metastatic disease or be under active surveillance prior to or after such treatment. The expected lifetime should be at least 4 months corresponding to a Karnofsky performance status above 60. They were also required to be able to read and understand Swedish. Patients fulfilling eligibility criteria were identified by the relevant teams of physicians at the two hospitals and invited to take part in the study. If interested, they were asked for informed consent by the study monitors.

### Trial design

This is a two-armed, prospective, open label, randomized, controlled intervention study. Patients were randomized to complete the EORTC-QLQ-C30 (hereafter referred to as EORTC) or the SEIQoL-DW (hereafter referred to as SEIQOL). The repeated use of these PROMs was defined as the study intervention. The EORTC arm was considered the control arm since previous studies had shown effects of an EORTC intervention [13]. A recent review has shown that the psychometric properties of individual measures, including the SEIQoL, are acceptable to use in a cancer population [27]. The EORTC is one of the most widely used and validated instruments in oncology and it is continuously being developed for example with regard to establishing minimally important differences (MID), normative data and developing computer adaptive testing (CAT) versions [30–33].

An independent study administrator randomly allocated patients to the two groups in blocks of 10. Patients filled out the respective PROMs prior to all their consultations at the clinic during a 4–6-month study period. Technical guidance was provided by either a nurse or a study monitor. Patients brought a printout of the responses to the consultation with the physician in order to support patient-physician communication. The patients usually had between 3 and 5 consultations during the follow-up period of 4–6 months. If actively treated with chemotherapy, patients usually met the physician at baseline, before cycle 2 and then after cycles 4, 8 etc. and after tumor evaluations every other month. If under surveillance, the control interval was usually every 2 months. Patients completed electronic versions of the SEIQoL or the EORTC on a touch screen computer in the waiting room before consultations. Patients were treated by two teams of oncologists, one at each center. In total, 15 oncologists agreed to engage in the study (6 in Uppsala, and 9 in Stockholm). All physicians provided both interventions, since they were too few to be effectively randomized.

All physicians attended a training session and an introductory session to learn about how to provide the intervention. They also had access to continuous support by the study monitors if needed. During the one-hour individual session, the researchers presented the study outline and presented the underlying philosophy and characteristics of both instruments. Special emphasis was placed on the interpretation of the QoL-results. The oncologist completed the instrument him or herself as a learning experience and patient cases were used to mimic the real-life situation. The oncologists were provided with a brief patient case and QoL-results and were asked to “think aloud” about how the results can be used to inform the consultation and clinical decision making.

#### Study outcomes and administration of evaluation questionnaires

All patients were asked to complete questionnaires after three of the consultations, i.e. at baseline, after about 2–3 months (after approximately 1–3 visits) and after 4–6 months (approximately 2–5 visits). Most questionnaires were completed at the hospital and some patients completed them at home. This was mainly due to fatigue and/or lack of time. Home completed questionnaires were, returned by post.

Instruments included were:

1. The Functional Assessment of Chronic Illness Therapy - Spiritual Well-Being (FACIT-Sp)
2. Global Quality of Life Uniscale (GQL-VAS)
3. Perceived quality of communication (PQC-VAS)
4. Medical Interview Satisfaction Scale (MISS-21)
5. Patient Global Impression of Change (PGIC)

### Measures

#### Demographic and clinical characteristics

Date of birth, gender, education and marital status were recorded at the baseline visit. Clinical characteristics, i.e. diagnosis and treatment at baseline and during the study period were collected from the medical records, including information on mortality 5 years after study ended.

#### Primary outcome

##### The functional assessment of chronic illness therapy - spiritual well-being (FACIT-Sp)

FACIT-Sp assesses HRQoL including spirituality. The FACIT-Sp is available in 11 languages allowing cross-cultural comparisons. It contains 39 items divided in five subscales: Physical well-being, Social/Family well-being, Emotional well-being, Functional well-being and Spiritual well-being. The measure was developed from the earlier version FACT (functional assessment of cancer therapy). The FACIT is a broader, more encompassing instrument. FACIT-questionnaires are being used in clinical trials and other treatment evaluations and are also used as intervention tools in the clinical management of symptoms etc. It is the most widely used measure of spiritual well-being among those with cancer and it is fit-for-purpose with regard to psychometric properties in this population [34–36].

The FACIT-Sp scoring guideline was used to derive a FACIT-Sp total score for each patient. The sum of the item scores was multiplied with the number of items in the subscale and then divided by the number of items answered to obtain the subscale score. The subscale scores were summed to derive the total score, which ranges from 0 to 156 points. Mean values for all patients’ results at baseline, second visit and 4–6 months’ visit, respectively, were used.

#### Secondary outcomes

##### Global quality of life uniscale (GQL-VAS)

Spitzer’s Uniscale is a single-item scale measuring patients’ global QoL. Patients were asked to place a mark on a horizontal 10 cm line indicating his/her QoL from very poor to very good. The wording was: “How would you rate your overall quality of life?” The instrument is valid and has been used in clinical trials. A visual analogue scale like the Uniscale is appropriate and effective, especially for patients who are seriously ill [37, 38]. The scale was converted to points between 0 and 100 (0 = very poor, 100 = very good). Mean values of patients’ results at baseline and 4–6 months visits were used.

##### Perceived quality of communication (PQC-VAS)

A VAS-scale was created by the researchers to measure the patient’ overall assessment of the quality of the patient-physician communication during the consultation. Patients were asked to place a mark on a horizontal 10 cm line ranging from very poor to very good. The scale was converted to points between 0 and 100 (0 = very poor, 100 = very good). Mean values of patients’ results at baseline and 4–6 months visit were used.

##### Medical interview satisfaction scale (MISS-21)

The ‘Medical Interview Satisfaction Scale’ was developed to analyze patient satisfaction with individual physician-patient consultations and has been shown to be reliable and valid [39]. It is a 21-statement survey divided into four subcategories: communication comfort (CC), distress relief (DR), compliance intent (CI), and rapport (R). It consists of a seven-point Likert scale, ranging from 1 (very strongly disagree) to 7 (very strongly agree). The maximum score is 147 indicating the highest possible satisfaction.

Patients indicated their level of agreement on the 7-point Likert scale with options: Very strongly disagree = 1, Strongly disagree = 2, Disagree = 3, Uncertain = 4, Strongly agree = 6 and Very strongly agree = 7. The subscales were summed and divided by number of items. The summary score was compiled by summing all items.

##### Patient global impression of change (PGIC)

The PGIC is a global measure indicating the degree of perceived change. In this study the change in question was in overall quality of life since the start of treatment. PGIC ratings are increasingly being used as a “gold standard” for determining clinically important change in measures such as ratings of pain [40]. It has also been used for ratings of pain in cancer populations [40, 41] and to determine important changes in HRQL in relation to the EORTC QLQ-C30 [42].

All patients were asked to complete the PGIC after every visit. The first question was: “How would you rate your quality of life now, compared to about two months ago?” Response options are: 1 = About the same, 2 = Better and 3 = Worse. If the patients chose option 2 or 3, they were referred to a 7-point Likert response scale with the following categories: 1 = “Very much”, 2 = “Much”, 3 = “Not so much”, 4 = “Moderate”, 5 = “Quite a bit”, 6 = “A little” and 7 = “Minimally”. Response options were combined to produce a single score ranging from − 7 (very much worse) to 7 (very much better).

### Statistical analyses

#### Power calculations

A superiority design was used since our hypothesis was that the individualized approach (SEIQoL) would be more effective than the standardized approach (EORTC), in achieving positive effects on primary and secondary study outcomes.

Based on calculations of clinically meaningful changes (MIDs) of the 7-graded FACIT-scale [43], 290 patients (145 patients per arm) were needed to detect a difference between arms, based on a significance level of 5% and a power of 80%. Based on earlier studies using the above inclusion criteria we estimated that 44% would be lost to follow up, hence we would need to include 518 patients in total, enabling detections of effect sizes (ES) of 0.33 or higher. Clinically meaningful changes for many PROMS have been reported to correspond to ES 0.20–0.50.

#### Descriptive statistics

**Fig. 1:**
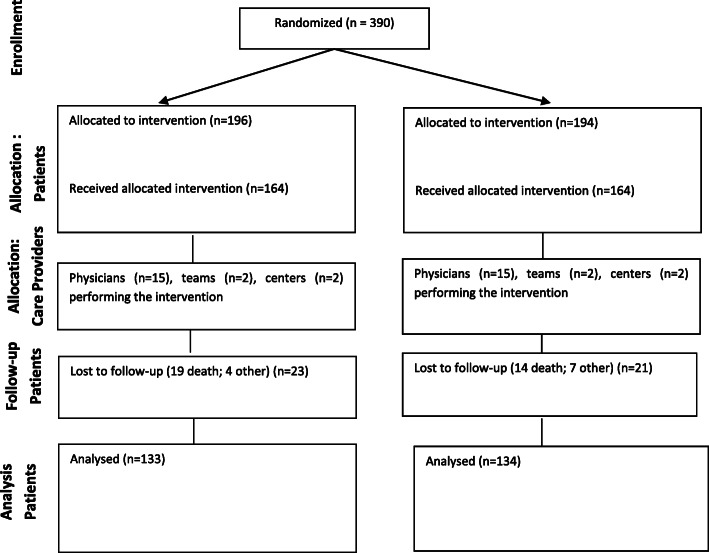
Consort diagram

The flow chart (Fig. 1) shows the number of patients from screening, inclusion and randomization, baseline assessment, second assessment and the final assessment after 4–6 months. Demographic information was compiled for all randomized patients.

##### Primary efficacy analysis

The presentation of the measurements/variables are based on a modified ITT analysis, including all randomized patients with, 1) information at baseline and, 2) at least one post-baseline value for the primary variable FACIT-Sp, and 3) and having been exposed to at least one intervention visit.

The null hypothesis is that there is no difference between EORTC and SEIQoL in the FACIT-Sp score post baseline. The alternative hypothesis is that there is a difference between groups. In order to test the hypothesis a linear mixed model with FACIT-Sp post-baseline scores as the dependent variable and group, time and FACIT-Sp baseline score as fixed covariates was used. Subjects were added as random factors in the model. The contrasts between groups are presented as a point estimate and the corresponding 95% two-sided confidence intervals of the difference. If the 95% confidence interval did not cover 0, the null hypothesis was rejected in favor of the alternative hypothesis.

##### Secondary efficacy analyses

The secondary endpoints: Global Quality of Life Uniscale (GQL-VAS), Perceived quality of communication (PQC-VAS), Medical Interview Satisfaction Scale (MISS-21) and Patient Global Impression of Change (PGIC) were analyzed using the same statistical model as the primary endpoint.

##### Survival analysis

Time from study baseline to death was analysed using Kaplan-Meier curves based on mortality date retrieved from the Swedish Cause of Death Register. The statistical test used to compare survival curves between the groups was a log rank test.

#### Statistical tests

All statistical tests were performed at a 5% significance level and presented using 95% confidence intervals. However, as no adjustment for multiplicity are done for the secondary endpoints, only *p*-value the primary endpoint can be interpreted as confirmatory. The *p*-values for secondary endpoints should thus be interpreted as exploratory.

## Results

### Patient characteristics

The flow of participants through the study is shown in Fig. 1. In total, 390 patients agreed to participate and were randomized and allocated to either EORTC or SEIQoL interventions. The first patient was randomized in September 2006 and the last one in December 2009. Of the 390 patients, 328 patients were recruited in Uppsala and 62 in Stockholm. In total, 267 were included in the analysis of the primary outcome, 133 patients completing the EORTC and 134 patients completing the SEIQOL (Fig. 1). The main reasons for attrition after randomization were patients not being treated i.e., receiving no exposure (EORTC: *n* = 32; SEIQOL: *n* = 30), and patients having no post-baseline visit (EORTC: *n* = 23; SEIQOL: *n* = 21). Of those who had no post-baseline visit, the majority were deceased (EORTC: *n* = 19; SEIQOL: *n* = 14), and the rest were lost to follow up (EORTC: *n* = 4; SEIQOL: *n* = 7).

Baseline characteristics of included patients are shown in Table 1. There were no major differences between patients being randomized and those who were included in the analyses regarding age, gender, family status, education, employment, diagnosis and treatment and there were no significant differences between the two analyzed groups (SEIQoL or EORTC) at baseline. Mean age was approximately 63 years. A majority of patients had university education. Nearly half of the patients were retired and a third were on sick leave. The main diagnosis was colorectal cancer and about one third had metastases. Most patients either had ongoing therapy (32%) or were planned to start on therapy at inclusion (59%). Nearly all patients received chemotherapy and, of these, about 10% received radiotherapy as well. One percent had radiotherapy only.

**Table 1:** Description of patients with at least one intervention

		EORTC (***N*** = 133)	SEIQOL (***N*** = 134)	Total (***N*** = 267)
**Gender**	n (%)			
**Men**		61 (45.9)	75 (56.0)	136 (51.0)
**Women**		72 (54.1)	59 (44.0)	131 (49.1)
**Age**	Mean (SD)	63.2 (9.5)	63.7 (9.6)	63.5 (9.5)
	Median	64	65	65
	Q1 & Q3	57 to 69	59 to 69	58 to 69
	n	133	134	267
**Family status**	n (%)			
**Living with parents/siblings**		0 (0.0)	0 (0.0)	0 (0.0)
**Single**		20 (16.0)	24 (19.2)	44 (17.6)
**Married**		84 (67.2)	78 (62.4)	162 (64.8)
**Cohabitant**		14 (11.2)	12 (9.6)	26 (10.4)
**Living apart together**		2 (1.6)	6 (4.8)	8 (3.2)
**Widow/widower**		5 (4.0)	5 (4.0)	10 (4.0)
**Highest education**	n (%)			
**Primary school (6 yrs)**		0 (0.0)	1 (2.7)	1 (1.3)
**Primary and lower secondary school (9 yrs)**		5 (12.5)	5 (13.5)	10 (13.0)
**Upper secondary education < 3 yrs**		6 (15.0)	3 (8.1)	9 (11.7)
**Upper secondary education > =3 yrs**		1 (2.5)	2 (5.4)	3 (3.9)
**Higher education < 3 yrs**		23 (57.5)	20 (54.0)	43 (55.8)
**Higher education > =3 yrs**		5 (12.5)	6 (16.2)	11 (14.3)
**Employment**	n (%)			
**Full-time**		10 (8.4)	11 (9.1)	21 (8.8)
**Part-time**		6 (5.0)	9 (7.4)	15 (6.2)
**Job seeking**		0 (0.0)	2 (1.6)	2 (0.8)
**Sick leave**		34 (28.6)	39 (32.2)	73 (30.4)
**Retired**		60 (50.4)	55 (45.4)	115 (47.9)
**Disability pension/ healthcare allowance**		8 (6.7)	5 (4.1)	13 (5.4)
**Other**		1 (0.8)	0 (0.0)	1 (0.4)
**Diagnosis**	n (%)			
**Gastric**		6 (4.8)	5 (4.0)	11 (4.4)
**Hepatobiliary**		5 (4.0)	4 (3.2)	9 (3.6)
**Liver (prim)**		1 (0.8)	0 (0.0)	1 (0.4)
**Pancreas**		19 (15.1)	18 (14.4)	37 (14.7)
**Colon**		51 (40.5)	63 (50.4)	114 (45.4)
**Rectum**		39 (31.0)	27 (21.6)	66 (26.3)
**Anus**		0 (0.0)	1 (0.8)	1 (0.4)
**Other/unknown GI**		5 (4.0)	7 (5.6)	12 (4.8)
**Treatment**	n (%)			
**Yes, ongoing at inclusion**		39 (32.0)	39 (32.0)	78 (32.0)
**Yes, starting at inclusion**		72 (59.0)	73 (59.8)	145 (59.4)
**No**		11 (9.0)	10 (8.2)	21 (8.6)
**Metastasis**	n (%)			
**Yes**		40 (32.3)	34 (27.9)	74 (30.1)
**No**		84 (67.7)	88 (72.1)	172 (69.9)
**Chemotherapy**	n (%)			
**Yes**		115 (98.3)	118 (99.2)	233 (98.7)
**No**		2 (1.7)	1 (0.8)	3 (1.3)
**Radiotherapy**	n (%)			
**Yes**		17 (12.8)	18 (13.4)	35 (13.1)
**No**		116 (87.2)	116 (86.6)	232 (86.9)
**Type of treatment**	n (%)			
**Chemotherapy**		100 (85.5)	104 (87.4)	204 (86.4)
**Radiotherapy with chemo**		15 (12.8)	14 (11.8)	29 (12.3)
**Radiotherapy without chemo**		2 (1.7)	1 (0.8)	3 (1.3)
**Frequency of exposure**	Mean (SD)	3.2 (0.8)	3.0 (0.8)	3.1 (0.8)
	Median	3	3	3
	Q1 & Q3	3 to 4	2 to 3	3 to 4
	n	133	134	267
**Days until 2nd questionnaire**	Mean (SD)	101.5 (67.3)	120.1 (84.5)	111.1 (77.0)
	Median	85	97	92
	Q1 & Q3	62 to 127	64 to 148	63 to 138
	n	120	127	247
**Days until 4 month questionnaire**	Mean (SD)	174.6 (62.6)	178.2 (79.8)	176.4 (71.4)
	Median	154	154	154
	Q1 & Q3	133 to 203	126 to 205	132 to 204
	n	97	95	192
**Survival**	n (%)			
**0–2 months**		0 (0.0)	0 (0.0)	0 (0.0)
**2–4 months**		5 (3.8)	2 (1.5)	7 (2.6)
**4–12 months**		27 (20.3)	28 (21.0)	55 (20.7)
**1–5 years**		62 (46.6)	65 (48.9)	127 (47.7)
**> 5 years**		10 (7.5)	7 (5.3)	17 (6.4)
**Still alive**		29 (21.8)	31 (23.3)	60 (22.6)

### Interventions and outcomes

After the baseline assessment, patients had a mean (and median) of 3 visits when they were exposed to either the EORTC or to the SEIQOL. The second assessment took place after median 92 days (EORTC: 85 days, Q1 = 62, Q3 = 127; SEIQoL: 97 days, Q1 = 64, Q3 = 148) and the final assessment after median 154 days for both EORTC (Q1 = 133, Q3 = 203) and SEIOQL (Q1 = 126, Q3 = 205).

**Fig. 2:**
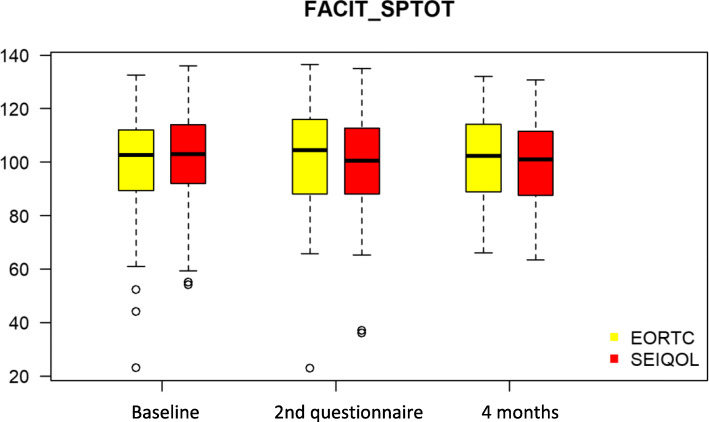
Boxplot Facit-Sp and EORTC versus SEIQOL-DW

The result of the comparison between the EORTC and the SEIQOL groups regarding the primary outcome (FACIT-Sp) is shown in Fig. 2. Detailed results for all outcomes are available in the Appendix. None of the differences between the two arms were statistically significant either at baseline, at the second assessment or the final assessment, and there were no mean changes over time. The mean FACIT-Sp score was about 100 over time for both arms. The GQL-VAS scale mean was about 66 over time for both arms combined. There was, however, a consistent difference between the two arms with EORTC having a lower mean, but again this difference was not statistically significant. The mean MISS-21 score was about 6 over time for both arms, and the mean PQC-VAS score was about 85. Patients reported a minimal improvement based on the PGIC at all assessment times in both groups.

**Fig. 3:**
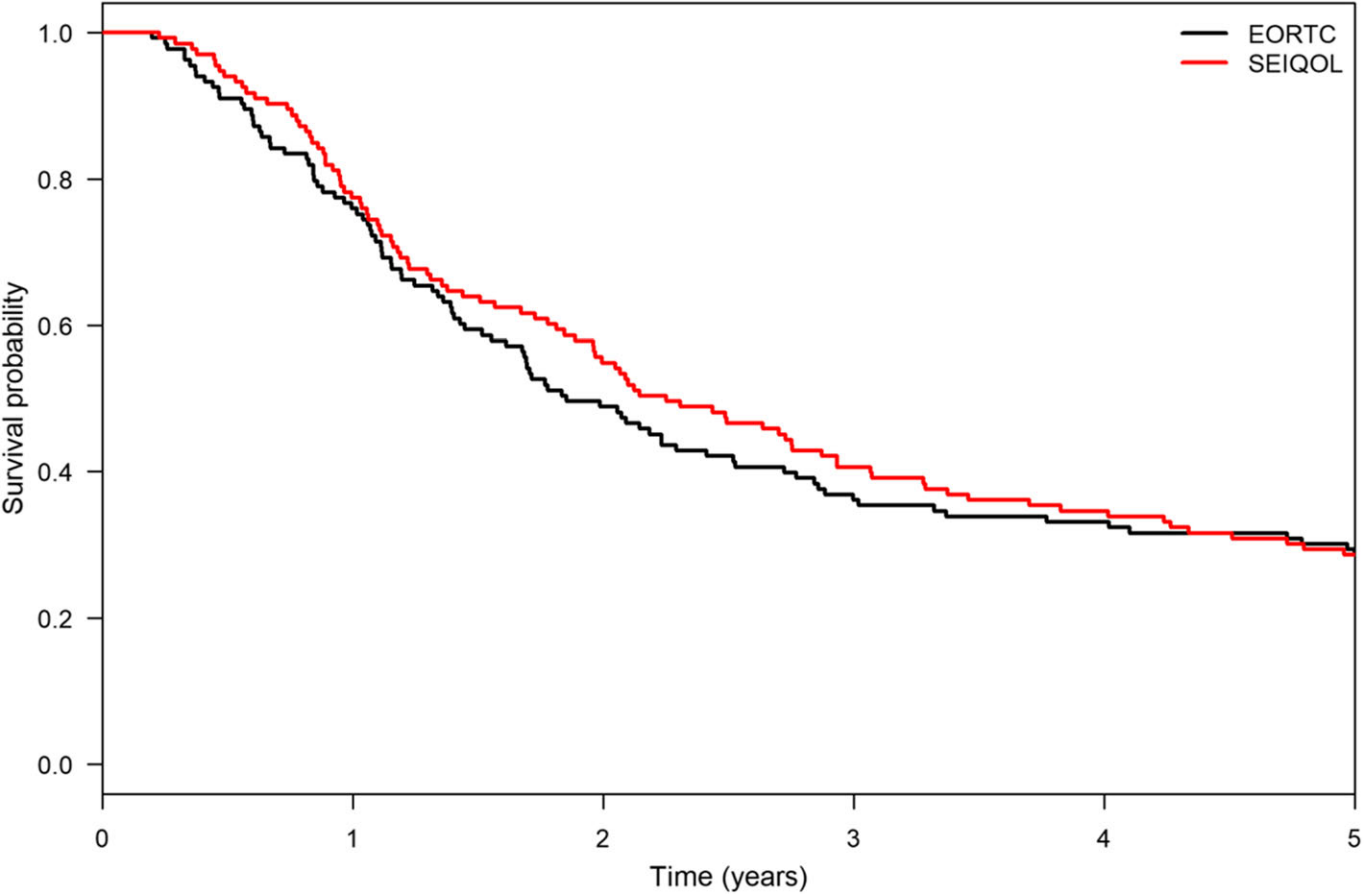
Kaplan-Meier curve for EORTC versus SEIQOL-DW

The survival analysis showed no difference between the two groups (Fig. 3). After 5 years, 23% of the analyzed patients were still alive. In total, almost 3% of the patients died within the first 4 months (EORTC: 3,8%; SEIQoL 1,5%), 21% died between 4 and 12 months (EORTC: 20%; SEIQoL 21%), and 48% between 1 and 5 years (EORTC: 47%; SEIQoL: 49%).

## Discussion

This study aimed to compare the effectiveness of two QoL instruments – the standardized instrument EORTC and the individualized instrument SEIQoL - in clinical practice. The individualized instrument did not show any superiority, there being no difference between the intervention groups. Neither of the instruments resulted in any changes in the study outcomes.

### Methodological considerations

The results of the study may reflect either a genuine lack of effect, sub-optimization of the intervention, or effects that were not possible to capture with the instruments used for assessing study outcomes. Given that the study compared a “new treatment” (SEIQOL) with a “standard treatment” (EORTC), and did not include any “no-intervention” arm, it cannot be ruled out that the instruments may still have favorably impacted on study outcomes. Theoretically, any deterioration of the patients’ QoL may have been slower, or patient satisfaction higher, than if no instrument had been used to support patient care. However, if the use of the PROMs in the present study had any impact at all, it is likely to be weaker than that found in the study by Velikova et al., where it was reported that EORTC-QOL-C30 had a slight positive impact on communication and on few QoL domains [13].

We based our power calculations on a large expected drop-out rate (44%) and decided to make a modified intention-to-treat (ITT) analysis, i.e. randomized patients with information at baseline and at least one intervention visit, prior to at least one post-baseline value for the primary variable FACIT-Sp, were included. Based on the literature, we assumed that the magnitude of effects from an intervention such as this would never be large enough to show up in a full ITT analysis, an assumption that also turned out to be true. We aimed for a power of 80% (i.e., 145 per arm) but ended up with 133 respectively 134 per arm. This is equivalent to a power of 76.5%, which we consider a nominal difference compared to 80% in the light of the lack of significant *p*-values with regard to the study outcomes. The study results are more likely due to lack of intervention effect than lack of power.

The are several reasons for the high drop-out rate. The intention was to include patients as soon as possible after referral to the oncology department, i.e. most patients were informed and asked for participation prior to their first visit at the out-patient unit when referred for oncologic neoadjuvant/adjuvant or palliative therapy, or continuous therapy initiated at a ward. For logistic reasons, only consultations at the out-patient ward were included, and patients requiring in-hospital care during the study period were lost. Furthermore, many referred patients do not start planned therapy after discussion with the oncologist, meaning they will not have any planned visits in the upcoming 4-month period. This means that they did not receive any exposure since they did not visit the department were administration of the SEIQoL/EORTC took place. In palliative situations, lack of response in disease progression within the first months is common in GI cancer patients, as is toxicity resulting in premature interruption of the treatment. If no adjuvant therapy, or only a short period of radiotherapy rather than chemoradiotherapy, is offered patients are referred back to the surgeon. Furthermore, if palliative therapy is no longer ongoing patients are often referred to palliative care units closer to their homes. Hence, GI cancer patients may not be ideal subjects for exploring the efficiency of an intervention aiming at improving the communication with the physician due to the variable disease course, often with poor prognosis and short survival. On the other hand, these vulnerable patients would benefit from an intervention improving their HRQoL. In this study, 3% of the patients died within the planned intervention period and 21% died between 4 and 12 months after baseline. In addition, a higher exposure to the intervention than the minimum two planned exposures might have been needed to achieve any impact on outcomes.

Performance bias must always be considered when blinding is not possible. Furthermore, since the same physicians provided both interventions, there may have been “cross-contamination”, i.e., physician behavior may have contributed to the lack of differences between the instruments.

Finally, the lack of statistically significant differences before and after the intervention may be due to a ceiling effect. The PQC- was 85%, and the MISS-21 score was 6/7 pre-intervention. Thus, most oncologists already had excellent communications skills at baseline.

### Discussion of the results

Other studies have shown that the use of PROMs increase symptom awareness, streamlines consultations, improves monitoring of treatment response and facilitates inter-professional communication [9, 10]. The impact on health outcomes is, however, less clear and more high-quality research is warranted [9–11]. A randomized controlled trial (RCT) showed that the use of EORTC-QOL-C30 before the consultation increased the patients’ and the physicians’ readiness to discuss QoL aspects on care and treatment [12]. Another RCT confirmed this and showed a positive impact on some QoL domains [13]. In yet another RCT on the impact of PROMs in routine oncology care, the health-related quality of life (HRQL) instrument FACT-G (Functional Assessment of Cancer Therapy-General) was used as the intervention tool [14]. No significant differences in HRQol or patient satisfaction were found between: 1) patients in usual care, 2) patients completing FACT-G assessment, and 3) patients completing FACT-G followed by a structured interview and discussion. Although, inconclusive, the above studies indicate that it is possible to achieve positive effects on communication and QoL using standardized QoL instruments in clinical practice, surpassing that of standard care, and we hypothesized that an individualized instrument would be even more effective in doing so. That turned out not to be the case.

Poor implementation may explain weak or non-existing effects of the PROMs on health outcomes. Known barriers to the use of PROMs in clinical practice include: technical problems hampering administration and completion; insufficient adoption by physicians due to lack of incentives or forgetfulness; use of PROMs that do not address matters that are important to the patient [9]. In order to improve implementation, there is a need to identify PROMs that patients find more acceptable, to increase training of physicians on PROM use (both in how to interpret scores, and how to act upon the results), to increase training of patients in the use of PROMs data for self-management. It is also necessary to limit data-collection to minimize patient burden, to provide alternative modes of data collection (e.g. web, telephone, tablet, or paper), to present patient data in easily accessible formats, to facilitate electronic transfer of data and to actively involve personnel, patients and physicians on site in order to minimize missing data [9, 44].

Several of the shortcomings listed above may well have contributed to the lack of detectable impact in the present study. Although SEIQoL-DW *does* address matters that are important to the patient – that is the signature strength of the instrument - there were problems regarding its administration and completion and there may have been insufficient adoption by physicians.

Many complex intervention trials have failed to show an effect and one plausible reason may be genuine ineffectiveness. It may also be due to inadequate intervention design and/or inadequate implementation [45]. Thus, it is important to optimize the intervention’s potential for effectiveness before embarking upon a RCT. Before launching this RCT, several feasibility studies were carried out [28, 29], and due attention was paid to the implementation process. Nevertheless, the intervention might still have been sub-optimal, especially with regard to the training of the physicians.

In light of empirically based recommendations made by Santana et al. [46], the outline of the training of physicians in this study had both strengths and weaknesses. On a positive note, experiential problem-based learning was used, and the training was brief and flexible and thus easy to fit into organizational practices. Some oncologists had participated in the pre-planning of the study, including the pilot studies, and had been able to raise any local concerns and influence the choice of PROs, graphic presentations of results etc. However, to the rest of the physicians, and all the patients, it was a case of fait accompli. Furthermore, the training did not take place in groups, there were no additional decision-support aids (e.g. on referral pathways). The physicians did not receive behavioral feedback after the training or during adoption and there was no training of other healthcare professionals such as nurses. Follow-up-sessions after training would have allowed for problems and barriers to implementation to be discussed – and collegial exchange of ideas and solutions to take place.

Although PROMs may facilitate the detection of, not least, psychosocial issues, this is only a first step. Physicians must also be ready to deal with the information that emerges. According to a Swedish study, most oncologists (93%) perceive one or more barriers in communicating about psychosocial aspects of cancer with patients [47]. On average, oncologists perceive five different communication barriers, which they themselves believe affect the consultation. These include insufficient consultation time, lack of resources for taking care of problems discovered, and lack of methods to evaluate patients’ psychosocial health. Less psychosocially oriented oncologists perceive more barriers, while oncologists with supplementary education with a psychosocial focus perceive fewer barriers. In conclusion, it may be that although the oncologist had a method to evaluate patients’ psychosocial health, they were not able to put the PROM results in effect due to all other barriers still being in place. If this would explain the lack of impact, then any introduction of PROMs in clinical practice should be preceded by supplementary training with a psychosocial focus. Furthermore, due attention must be paid to the preconditions for the consultations, e.g. consultation time, and possibilities to refer patients to colleagues that are specialized on psychosocial concerns.

It could also be that PROMs are only useful if used by oncologists and patients who are convinced that they contribute meaningful information to the consultation. If such intrinsic motivation exists, the results will probably be used more actively and this in turn might influence clinical decisions and thereby improve outcomes. Thus, physician and patient ownership may be key success factors. In order for physicians and other health care professionals to be committed to the use of PROMs, the entire health care team has to be actively involved in designing the intervention, and adapting it to the work flow at the clinic. Patients should also be trained in the use of PROMs for self-management purposes.

It could also be that devising *either* a standardized or an individualized PROM is a wrong approach in itself. Perhaps different PROMs should be seen as complementary tools in the physician’s communication toolbox, to be employed according to the situation and individual physician and patient preferences. Individualized PROMs may have their chief value as ‘conversation openers’, prompting patients to describe their situation in their own words, although their dynamic nature also means that they may be more difficult to use in follow-up [17]. Standardized instruments, on the other hand, may be more effective in eliciting important information from patients who are less willing to contribute information in their own words, and may be easier to use in follow-up studies and in aggregating data (unless physicians tamper with them in order to increase their clinical usefulness) [17, 48].

## Conclusion

The great complexity of using PROMs in clinical practice may be discouraging, but the knowledge base for their effective use is growing. A recent example is the “PRO-cision Medicine Methods Tool kit” paper series in Medical Care, which aims at simplifying the interpretation of PRO scores and facilitating action based on PRO result [49].

This study did not find any differences between the standardized instrument EORTC and the individual instrument SEIQoL. This may be explained by inadequate implementation. In order to generate a definitive view on the effects of different PROMs’ in monitoring and improving patients’ QoL, further studies are required with a major focus on the implementation process. The possible effects of situational use of both types of PROMs by the same physician should also be explored.

## Data Availability

The data that support the findings of this study are available from the corresponding author, [LR], upon reasonable request.

## Appendix

**Table 2:** Outcomes^a^ over time for analyzed patients

	EORTC (***N*** = 133)	SEIQOL (***N*** = 134)	Total (***N*** = 267)	***P***-value
**FACIT-SP Total**				
**Baseline**				
Mean (SD)	100.7 (17.4)	102.4 (17.0)	101.6 (17.2)	0.396
Median	103.7	101.8	103	
Q1 & Q3	92 to 113	92 to 114.2	92 to 113.9	
n	133	134	267	
**2nd questionnaire**				
Mean (SD)	101.8 (18.4)	100.6 (17.4)	101.2 (17.9)	0.588
Median	104.5	100.5	101.8	
Q1 & Q3	88.5 to 115.9	88 to 112.7	88 to 115	
n	126	130	256	
**4 months**				
Mean (SD)	101.6 (16.3)	99.1 (16.6)	100.4 (16.5)	0.289
Median	102.3	101.1	102	
Q1 & Q3	88.8 to 114.2	87.5 to 111.4	88.1 to 113	
n	101	98	199	
**FACIT PWB**				
**Baseline**				
Mean (SD)	21.0 (5.3)	20.9 (5.6)	20.9 (5.4)	0.959
Median	22	22	22	
Q1 & Q3	18 to 25	17 to 25.7	18 to 25	
n	131	133	264	
**2nd questionnaire**				
Mean (SD)	20.6 (5.9)	20.3 (5.1)	20.4 (5.5)	0.678
Median	22	21	21	
Q1 & Q3	17 to 25	16 to 24.1	17 to 25	
n	125	128	253	
**4 months**				
Mean (SD)	20.0 (5.9)	19.6 (5.8)	19.8 (5.8)	0.689
Median	21	21	21	
Q1 & Q3	16 to 25	16 to 24	16 to 25	
n	101	98	199	
**FACIT SWB**				
**Baseline**				
Mean (SD)	23.8 (3.5)	23.5 (3.7)	23.6 (3.6)	0.402
Median	24	24	24	
Q1 & Q3	22 to 26.8	21 to 26	21 to 26.4	
n	130	133	263	
**2nd questionnaire**				
Mean (SD)	23.6 (4.5)	22.6 (3.7)	23.1 (4.2)	0.074
Median	25	23	24	
Q1 & Q3	21 to 26.8	20 to 25.3	21 to 26	
n	125	128	253	
**4 months**				
Mean (SD)	23.2 (4.2)	22.7 (4.5)	23.0 (4.3)	0.413
Median	24	24	24	
Q1 & Q3	21 to 26.8	21 to 25.7	21 to 26	
n	101	98	199	
**FACIT EWB**				
**Baseline**				
Mean (SD)	6.6 (4.6)	6.3 (4.3)	6.5 (4.4)	0.634
Median	5	6	5	
Q1 & Q3	3 to 9.2	3 to 9	3 to 9	
n	132	133	265	
**2nd questionnaire**				
Mean (SD)	5.6 (4.5)	5.9 (4.7)	5.7 (4.6)	0.566
Median	5	5	5	
Q1 & Q3	2 to 8	2 to 9.2	2 to 9	
n	125	128	253	
**4 months**				
Mean (SD)	6.3 (5.0)	6.7 (4.9)	6.5 (5.0)	0.578
Median	5	6	5	
Q1 & Q3	2 to 9	3 to 9.8	3 to 9	
n	101	98	199	
**FACIT FWB**				
**Baseline**				
Mean (SD)	17.3 (5.4)	18.2 (5.8)	17.8 (5.6)	0.172
Median	18	19	19	
Q1 & Q3	14.8 to 21	14 to 23	14 to 22	
n	132	133	265	
**2nd questionnaire**				
Mean (SD)	17.9 (5.9)	17.7 (5.7)	17.8 (5.8)	0.724
Median	19	18	18	
Q1 & Q3	13 to 23	14 to 21	13 to 22	
n	125	128	253	
**4 months**				
Mean (SD)	17.4 (6.1)	17.2 (5.6)	17.3 (5.8)	0.785
Median	18	18	18	
Q1 & Q3	13 to 22	14 to 21	13 to 21	
n	101	98	199	
**FACIT SPS**				
**Baseline**				
Mean (SD)	33.5 (8.0)	35.1 (8.5)	34.3 (8.3)	0.132
Median	33.3	36	34.7	
Q1 & Q3	28 to 39.7	29.3 to 41.3	29 to 41	
n	131	130	261	
**2nd questionnaire**				
Mean (SD)	35.0 (9.1)	35.1 (8.2)	35.0 (8.6)	0.898
Median	37	36	36	
Q1 & Q3	28 to 42.7	29.6 to 41.8	28 to 42	
n	125	130	255	
**4 months**				
Mean (SD)	34.7 (8.4)	33.6 (8.9)	34.2 (8.7)	0.363
Median	36	34.2	35	
Q1 & Q3	28 to 41.3	26.7 to 40.2	27 to 41.1	
n	101	96	197	
**FACIT FG Total**				
**Baseline**				
Mean (SD)	68.1 (11.4)	68.4 (10.0)	68.3 (10.7)	0.830
Median	69	69.8	69.2	
Q1 & Q3	62 to 76.7	61.7 to 76	62 to 76	
n	132	134	266	
**2nd questionnaire**				
Mean (SD)	67.6 (10.9)	66.5 (10.3)	67.1 (10.6)	0.392
Median	68.2	67.2	68	
Q1 & Q3	60 to 76	59.7 to 75	59.8 to 75.8	
n	125	128	253	
**4 months**				
Mean (SD)	66.9 (11.1)	66.2 (9.8)	66.5 (10.5)	0.653
Median	69	66.2	67	
Q1 & Q3	59 to 75.3	60 to 74	59 to 74.8	
n	101	98	199	
**MISS-21 Total**				
**Baseline**				
Mean (SD)	5.9 (0.9)	6.0 (0.8)	5.9 (0.8)	0.422
Median	6.1	6.2	6.1	
Q1 & Q3	5.4 to 6.6	5.4 to 6.6	5.4 to 6.6	
n	133	134	267	
**2nd questionnaire**				
Mean (SD)	6.0 (0.8)	5.9 (0.8)	6.0 (0.8)	0.402
Median	6.2	6.1	6.1	
Q1 & Q3	5.6 to 6.6	5.5 to 6.6	5.6 to 6.6	
n	127	131	258	
**4 months**				
Mean (SD)	5.9 (1.0)	5.9 (0.8)	5.9 (0.9)	0.807
Median	6.3	6.1	6.2	
Q1 & Q3	5.4 to 6.7	5.5 to 6.5	5.4 to 6.6	
n	101	98	199	
**MISS-21 Distress Relief**				
**Baseline**				
Mean (SD)	5.4 (1.3)	5.5 (1.3)	5.4 (1.3)	0.900
Median	5.7	5.8	5.8	
Q1 & Q3	4.8 to 6.3	4.8 to 6.3	4.8 to 6.3	
n	132	134	266	
**2nd questionnaire**				
Mean (SD)	5.5 (1.3)	5.4 (1.2)	5.5 (1.3)	0.620
Median	5.7	5.7	5.7	
Q1 & Q3	5 to 6.5	4.7 to 6.5	4.8 to 6.5	
n	127	130	257	
**4 months**				
Mean (SD)	5.6 (1.4)	5.5 (1.2)	5.5 (1.3)	0.582
Median	6	5.8	5.8	
Q1 & Q3	5 to 6.7	4.8 to 6.2	4.8 to 6.6	
n	101	98	199	
**MISS-21 Communciation Comfort**				
**Baseline**				
Mean (SD)	6.0 (1.2)	6.1 (0.9)	6.0 (1.0)	0.413
Median	6.2	6.2	6.2	
Q1 & Q3	5.5 to 7	5.5 to 7	5.5 to 7	
n	133	134	267	
**2nd questionnaire**				
Mean (SD)	6.1 (1.0)	6.1 (0.9)	6.1 (1.0)	0.975
Median	6.5	6.2	6.5	
Q1 & Q3	5.5 to 7	5.5 to 7	5.5 to 7	
n	127	131	258	
**4 months**				
Mean (SD)	5.9 (1.2)	6.1 (1.0)	6.0 (1.1)	0.243
Median	6.2	6.5	6.2	
Q1 & Q3	5.5 to 7	5.5 to 7	5.5 to 7	
n	101	98	199	
**MISS-21 Rapport**				
**Baseline**				
Mean (SD)	6.1 (1.0)	6.2 (0.9)	6.2 (0.9)	0.648
Median	6.5	6.4	6.5	
Q1 & Q3	5.8 to 6.9	5.9 to 6.9	5.8 to 6.9	
n	133	134	267	
**2nd questionnaire**				
Mean (SD)	6.3 (0.9)	6.1 (1.0)	6.2 (0.9)	0.129
Median	6.6	6.4	6.6	
Q1 & Q3	5.9 to 7	5.8 to 6.9	5.9 to 6.9	
n	127	131	258	
**4 months**				
Mean (SD)	6.2 (1.2)	6.1 (1.0)	6.1 (1.1)	0.538
Median	6.8	6.2	6.5	
Q1 & Q3	5.9 to 7	5.9 to 6.9	5.9 to 7	
n	101	98	199	
**MISS-21 Compliance Intent**				
**Baseline**				
Mean (SD)	6.0 (1.1)	6.3 (0.9)	6.2 (1.0)	0.073
Median	6.4	6.7	6.7	
Q1 & Q3	5.3 to 7	6 to 7	5.7 to 7	
n	130	132	262	
**2nd questionnaire**				
Mean (SD)	6.1 (1.2)	6.1 (1.1)	6.1 (1.2)	0.991
Median	6.7	6.3	6.4	
Q1 & Q3	5.3 to 7	5.3 to 7	5.3 to 7	
n	125	129	254	
**4 months**				
Mean (SD)	6.0 (1.3)	6.0 (1.1)	6.0 (1.2)	0.782
Median	6.3	6.3	6.3	
Q1 & Q3	5 to 7	5 to 7	5 to 7	
n	100	96	196	
**Perceived Quality of Communication (PQC VAS)**				
**Baseline**				
Mean (SD)	85.3 (15.4)	85.7 (13.4)	85.5 (14.4)	0.794
Median	89.5	90	90	
Q1 & Q3	81.8 to 96	78.5 to 96	80 to 96	
n	128	127	255	
**2nd questionnaire**				
Mean (SD)	86.1 (14.3)	85.6 (16.7)	85.8 (15.5)	0.794
Median	90	91	91	
Q1 & Q3	81.5 to 97	80 to 96	80.2 to 96.8	
n	123	127	250	
**4 months**				
Mean (SD)	83.2 (19.0)	84.9 (13.6)	84.0 (16.5)	0.481
Median	90.5	87.5	89	
Q1 & Q3	78.8 to 96	78 to 95	78 to 96	
n	96	94	190	
**Global Quality of Life Uniscale (GQL VAS)**				
**Baseline**				
Mean (SD)	63.3 (21.5)	68.2 (21.2)	65.8 (21.4)	0.062
Median	68	71	70	
Q1 & Q3	49 to 79	53.8 to 84	50 to 82	
n	129	132	261	
**2nd questionnaire**				
Mean (SD)	65.3 (23.3)	67.7 (21.9)	66.5 (22.6)	0.394
Median	70	71	70	
Q1 & Q3	49 to 83	51.5 to 84.8	50 to 83.5	
n	125	130	255	
**4 months**				
Mean (SD)	64.3 (24.8)	66.7 (21.9)	65.5 (23.4)	0.469
Median	71	71	71	
Q1 & Q3	47 to 82	54 to 82	49.8 to 82	
n	101	95	196	
**PGIC 1 – Overall QoL change**				
**Baseline**				
Mean (SD)	0.1 (3.7)	0.8 (3.8)	0.4 (3.8)	0.128
Median	0	0	0	
Q1 & Q3	-3 to 4	0 to 4.8	0 to 4	
n	132	130	262	
**2nd questionnaire**				
Mean (SD)	0.6 (3.5)	0.8 (3.3)	0.7 (3.4)	0.591
Median	0	0	0	
Q1 & Q3	0 to 4	0 to 4	0 to 4	
n	125	129	254	
**4 months**				
Mean (SD)	0.2 (3.5)	0.7 (3.4)	0.4 (3.4)	0.265
Median	0	0	0	
Q1 & Q3	0 to 2	0 to 4	0 to 4	
n	101	98	199	
**MISS-21 item 1**				
**Baseline**				
Mean (SD)	5.5 (1.4)	5.3 (1.5)	5.4 (1.5)	0.253
Median	6	6	6	
Q1 & Q3	5 to 7	4 to 7	4 to 7	
n	125	126	251	
**2nd questionnaire**				
Mean (SD)	5.3 (1.7)	5.2 (1.6)	5.3 (1.6)	0.682
Median	6	6	6	
Q1 & Q3	4 to 7	4.5 to 6	4 to 7	
n	121	123	244	
**4 months**				
Mean (SD)	5.7 (1.4)	5.3 (1.5)	5.5 (1.5)	0.077
Median	6	6	6	
Q1 & Q3	5 to 7	4 to 6	4 to 7	
n	98	93	191	
**MISS-21 item 2**				
**Baseline**				
Mean (SD)	5.5 (1.6)	5.5 (1.8)	5.5 (1.7)	0.981
Median	6	6	6	
Q1 & Q3	5 to 7	4 to 7	4 to 7	
n	126	129	255	
**2nd questionnaire**				
Mean (SD)	5.5 (1.7)	5.5 (1.7)	5.5 (1.7)	0.989
Median	6	6	6	
Q1 & Q3	4.5 to 7	5 to 7	5 to 7	
n	123	120	243	
**4 months**				
Mean (SD)	5.5 (1.8)	5.6 (1.6)	5.5 (1.7)	0.891
Median	6	6	6	
Q1 & Q3	4.2 to 7	5 to 7	5 to 7	
n	98	94	192	
**MISS-21 item 3**				
**Baseline**				
Mean (SD)	5.6 (1.7)	5.5 (1.8)	5.5 (1.7)	0.652
Median	6	6	6	
Q1 & Q3	5 to 7	4 to 7	5 to 7	
n	130	127	257	
**2nd questionnaire**				
Mean (SD)	5.7 (1.6)	5.5 (1.7)	5.6 (1.7)	0.482
Median	6	6	6	
Q1 & Q3	5 to 7	4 to 7	5 to 7	
n	125	119	244	
**4 months**				
Mean (SD)	5.8 (1.6)	5.7 (1.5)	5.7 (1.5)	0.648
Median	6	6	6	
Q1 & Q3	5 to 7	5 to 7	5 to 7	
n	97	94	191	
**MISS-21 item 4**				
**Baseline**				
Mean (SD)	3.4 (2.4)	3.4 (2.4)	3.4 (2.4)	0.901
Median	2	2	2	
Q1 & Q3	1 to 6	1 to 6	1 to 6	
n	127	125	252	
**2nd questionnaire**				
Mean (SD)	2.7 (2.2)	3.1 (2.2)	2.9 (2.2)	0.261
Median	2	2	2	
Q1 & Q3	1 to 4.5	1 to 5	1 to 5	
n	123	124	247	
**4 months**				
Mean (SD)	3.3 (2.4)	3.0 (2.2)	3.2 (2.3)	0.262
Median	2	2	2	
Q1 & Q3	1 to 6	1 to 4	1 to 6	
n	99	93	192	
**MISS-21 item 5**				
**Baseline**				
Mean (SD)	5.1 (1.8)	5.2 (1.7)	5.1 (1.8)	0.626
Median	6	6	6	
Q1 & Q3	4 to 7	4 to 7	4 to 7	
n	126	128	254	
**2nd questionnaire**				
Mean (SD)	5.2 (1.7)	5.1 (1.8)	5.1 (1.7)	0.727
Median	6	6	6	
Q1 & Q3	4 to 6.8	4 to 7	4 to 7	
n	122	118	240	
**4 months**				
Mean (SD)	5.5 (1.7)	5.4 (1.6)	5.5 (1.6)	0.489
Median	6	6	6	
Q1 & Q3	5 to 7	5 to 7	5 to 7	
n	98	95	193	
**MISS-21 item 6**				
**Baseline**				
Mean (SD)	5.7 (1.4)	5.7 (1.4)	5.7 (1.4)	0.945
Median	6	6	6	
Q1 & Q3	5 to 7	5 to 7	5 to 7	
n	131	133	264	
**2nd questionnaire**				
Mean (SD)	5.8 (1.4)	5.7 (1.4)	5.8 (1.4)	0.338
Median	6	6	6	
Q1 & Q3	5 to 7	5 to 7	5 to 7	
n	127	129	256	
**4 months**				
Mean (SD)	6.0 (1.4)	5.6 (1.5)	5.8 (1.4)	0.064
Median	7	6	6	
Q1 & Q3	5 to 7	5 to 7	5 to 7	
n	100	97	197	
**MISS-21 item 7**				
**Baseline**				
Mean (SD)	6.3 (1.2)	6.3 (1.0)	6.3 (1.1)	0.745
Median	7	7	7	
Q1 & Q3	6 to 7	6 to 7	6 to 7	
n	132	133	265	
**2nd questionnaire**				
Mean (SD)	6.4 (1.0)	6.3 (1.1)	6.4 (1.0)	0.484
Median	7	7	7	
Q1 & Q3	6 to 7	6 to 7	6 to 7	
n	126	130	256	
**4 months**				
Mean (SD)	6.4 (1.1)	6.2 (1.1)	6.3 (1.1)	0.441
Median	7	7	7	
Q1 & Q3	6 to 7	6 to 7	6 to 7	
n	100	96	196	
**MISS-21 item 8**				
**Baseline**				
Mean (SD)	6.2 (1.2)	6.4 (1.0)	6.3 (1.1)	0.154
Median	7	7	7	
Q1 & Q3	6 to 7	6 to 7	6 to 7	
n	132	133	265	
**2nd questionnaire**				
Mean (SD)	6.5 (0.8)	6.3 (1.1)	6.4 (1.0)	0.096
Median	7	7	7	
Q1 & Q3	6 to 7	6 to 7	6 to 7	
n	127	129	256	
**4 months**				
Mean (SD)	6.3 (1.3)	6.3 (1.0)	6.3 (1.2)	0.865
Median	7	7	7	
Q1 & Q3	6 to 7	6 to 7	6 to 7	
n	100	97	197	
**MISS-21 item 9**				
**Baseline**				
Mean (SD)	1.4 (1.3)	1.2 (0.7)	1.3 (1.1)	0.034
Median	1	1	1	
Q1 & Q3	1 to 1	1 to 1	1 to 1	
n	132	133	265	
**2nd questionnaire**				
Mean (SD)	1.3 (1.0)	1.2 (0.5)	1.2 (0.8)	0.244
Median	1	1	1	
Q1 & Q3	1 to 1	1 to 1	1 to 1	
n	127	129	256	
**4 months**				
Mean (SD)	1.4 (1.2)	1.2 (0.7)	1.3 (1.0)	0.103
Median	1	1	1	
Q1 & Q3	1 to 1	1 to 1	1 to 1	
n	100	97	197	
**MISS-21 item 10**				
**Baseline**				
Mean (SD)	6.1 (1.4)	6.0 (1.4)	6.0 (1.4)	0.506
Median	7	6	7	
Q1 & Q3	6 to 7	6 to 7	6 to 7	
n	131	133	264	
**2nd questionnaire**				
Mean (SD)	6.1 (1.4)	5.9 (1.5)	6.0 (1.5)	0.187
Median	7	6	7	
Q1 & Q3	6 to 7	5 to 7	6 to 7	
n	126	129	255	
**4 months**				
Mean (SD)	6.0 (1.6)	5.8 (1.5)	5.9 (1.5)	0.474
Median	7	6	6	
Q1 & Q3	6 to 7	5 to 7	5 to 7	
n	98	96	194	
**MISS-21 item 11**				
**Baseline**				
Mean (SD)	6.3 (1.2)	6.3 (1.1)	6.3 (1.2)	0.927
Median	7	7	7	
Q1 & Q3	6 to 7	6 to 7	6 to 7	
n	131	132	263	
**2nd questionnaire**				
Mean (SD)	6.4 (1.1)	6.2 (1.2)	6.3 (1.1)	0.257
Median	7	7	7	
Q1 & Q3	6 to 7	6 to 7	6 to 7	
n	127	130	257	
**4 months**				
Mean (SD)	6.1 (1.5)	6.1 (1.3)	6.1 (1.4)	0.933
Median	7	7	7	
Q1 & Q3	6 to 7	6 to 7	6 to 7	
n	100	94	194	
**MISS-21 item 12**				
**Baseline**				
Mean (SD)	6.1 (1.2)	6.2 (0.9)	6.2 (1.1)	0.613
Median	6	6	6	
Q1 & Q3	6 to 7	6 to 7	6 to 7	
n	131	133	264	
**2nd questionnaire**				
Mean (SD)	6.3 (1.0)	6.1 (1.2)	6.2 (1.1)	0.046
Median	7	6	7	
Q1 & Q3	6 to 7	6 to 7	6 to 7	
n	127	128	255	
**4 months**				
Mean (SD)	6.0 (1.5)	6.0 (1.2)	6.0 (1.4)	0.757
Median	7	6	6	
Q1 & Q3	6 to 7	6 to 7	6 to 7	
n	100	97	197	
**MISS-21 item 13**				
**Baseline**				
Mean (SD)	1.9 (1.9)	1.9 (1.7)	1.9 (1.8)	0.874
Median	1	1	1	
Q1 & Q3	1 to 2	1 to 2	1 to 2	
n	129	133	262	
**2nd questionnaire**				
Mean (SD)	2.2 (2.1)	1.9 (1.7)	2.0 (1.9)	0.338
Median	1	1	1	
Q1 & Q3	1 to 2	1 to 2	1 to 2	
n	127	129	256	
**4 months**				
Mean (SD)	2.1 (1.9)	2.0 (1.9)	2.0 (1.9)	0.738
Median	1	1	1	
Q1 & Q3	1 to 2	1 to 2	1 to 2	
n	101	97	198	
**MISS-21 item 14**				
**Baseline**				
Mean (SD)	1.4 (1.2)	1.3 (1.1)	1.4 (1.2)	0.927
Median	1	1	1	
Q1 & Q3	1 to 1	1 to 1	1 to 1	
n	128	130	258	
**2nd questionnaire**				
Mean (SD)	1.3 (1.2)	1.4 (1.2)	1.4 (1.2)	0.641
Median	1	1	1	
Q1 & Q3	1 to 1	1 to 1	1 to 1	
n	122	128	250	
**4 months**				
Mean (SD)	1.5 (1.3)	1.5 (1.2)	1.5 (1.3)	0.934
Median	1	1	1	
Q1 & Q3	1 to 1	1 to 1	1 to 1	
n	98	97	195	
**MISS-21 item 15**				
**Baseline**				
Mean (SD)	6.1 (1.3)	6.2 (1.1)	6.1 (1.2)	0.411
Median	7	7	7	
Q1 & Q3	6 to 7	6 to 7	6 to 7	
n	128	134	262	
**2nd questionnaire**				
Mean (SD)	6.3 (1.1)	6.2 (1.3)	6.3 (1.2)	0.359
Median	7	7	7	
Q1 & Q3	6 to 7	6 to 7	6 to 7	
n	125	131	256	
**4 months**				
Mean (SD)	6.2 (1.4)	6.2 (1.1)	6.2 (1.2)	0.998
Median	7	7	7	
Q1 & Q3	6 to 7	6 to 7	6 to 7	
n	100	96	196	
**MISS-21 item 16**				
**Baseline**				
Mean (SD)	6.4 (0.9)	6.5 (0.9)	6.4 (0.9)	0.652
Median	7	7	7	
Q1 & Q3	6 to 7	6 to 7	6 to 7	
n	128	133	261	
**2nd questionnaire**				
Mean (SD)	6.6 (0.9)	6.4 (1.1)	6.5 (1.0)	0.139
Median	7	7	7	
Q1 & Q3	6 to 7	6 to 7	6 to 7	
n	125	131	256	
**4 months**				
Mean (SD)	6.4 (1.1)	6.3 (1.0)	6.4 (1.1)	0.746
Median	7	7	7	
Q1 & Q3	6 to 7	6 to 7	6 to 7	
n	100	97	197	
**MISS-21 item 17**				
**Baseline**				
Mean (SD)	5.1 (1.7)	5.2 (1.6)	5.2 (1.6)	0.524
Median	5	6	5	
Q1 & Q3	4 to 7	4 to 7	4 to 7	
n	127	128	255	
**2nd questionnaire**				
Mean (SD)	5.4 (1.7)	5.3 (1.5)	5.3 (1.6)	0.760
Median	6	6	6	
Q1 & Q3	5 to 7	4 to 7	4 to 7	
n	118	126	244	
**4 months**				
Mean (SD)	5.2 (1.8)	5.1 (1.6)	5.2 (1.7)	0.536
Median	6	5	6	
Q1 & Q3	4 to 7	4 to 6	4 to 7	
n	97	94	191	
**MISS-21 item 18**				
**Baseline**				
Mean (SD)	5.9 (1.3)	6.0 (1.2)	5.9 (1.2)	0.453
Median	6	6	6	
Q1 & Q3	5 to 7	6 to 7	5 to 7	
n	129	129	258	
**2nd questionnaire**				
Mean (SD)	6.0 (1.3)	5.9 (1.3)	6.0 (1.3)	0.328
Median	6	6	6	
Q1 & Q3	6 to 7	5 to 7	5.5 to 7	
n	125	126	251	
**4 months**				
Mean (SD)	5.8 (1.4)	5.7 (1.3)	5.8 (1.4)	0.748
Median	6	6	6	
Q1 & Q3	5 to 7	5 to 7	5 to 7	
n	100	95	195	
**MISS-21 item 19**				
**Baseline**				
Mean (SD)	6.1 (1.2)	6.3 (1.0)	6.2 (1.1)	0.264
Median	7	7	7	
Q1 & Q3	6 to 7	6 to 7	6 to 7	
n	125	129	254	
**2nd questionnaire**				
Mean (SD)	6.2 (1.2)	6.0 (1.3)	6.1 (1.3)	0.258
Median	7	6	7	
Q1 & Q3	6 to 7	6 to 7	6 to 7	
n	123	128	251	
**4 months**				
Mean (SD)	6.0 (1.4)	5.8 (1.3)	5.9 (1.3)	0.463
Median	7	6	6	
Q1 & Q3	5 to 7	5 to 7	5 to 7	
n	100	93	193	
**MISS-21 item 20**				
**Baseline**				
Mean (SD)	2.1 (1.7)	1.7 (1.3)	1.9 (1.5)	0.035
Median	1	1	1	
Q1 & Q3	1 to 2	1 to 2	1 to 2	
n	127	130	257	
**2nd questionnaire**				
Mean (SD)	2.0 (1.7)	2.0 (1.5)	2.0 (1.6)	0.935
Median	1	1	1	
Q1 & Q3	1 to 2	1 to 2	1 to 2	
n	122	127	249	
**4 months**				
Mean (SD)	2.0 (1.6)	2.1 (1.7)	2.1 (1.6)	0.715
Median	1	1	1	
Q1 & Q3	1 to 2	1 to 2.5	1 to 2	
n	99	95	194	
**MISS-21 item 21**				
**Baseline**				
Mean (SD)	1.8 (1.5)	1.8 (1.5)	1.8 (1.5)	0.824
Median	1	1	1	
Q1 & Q3	1 to 2	1 to 2	1 to 2	
n	129	127	256	
**2nd questionnaire**				
Mean (SD)	2.0 (1.8)	1.8 (1.4)	1.9 (1.6)	0.330
Median	1	1	1	
Q1 & Q3	1 to 2	1 to 2	1 to 2	
n	122	126	248	
**4 months**				
Mean (SD)	2.0 (1.6)	1.7 (1.4)	1.8 (1.5)	0.272
Median	1	1	1	
Q1 & Q3	1 to 2	1 to 2	1 to 2	
n	98	96	194	

^a^)

*FACIT-Sp* The Functional Assessment of Chronic Illness Therapy - Spiritual Well-Being

GQL-VAS Global Quality of Life Uniscale

*PQC-VAS* Perceived Quality of Communication

*MISS-21* Medical Interview Satisfaction Scale

*PGIC* Patient Global Impression of Change

## Abbreviations

EORTC-QOL-C30: European Organization for Research and Treatment of Cancer Quality of Life C-30
FACIT-Sp: The Functional Assessment of Chronic Illness Therapy - Spiritual Well-Being
FACT: Functional assessment of cancer therapy
FACT-G: Functional assessment of cancer therapy-general
GI: Gastrointestinal
GQL-VAS: Global Quality of Life Uniscale
HRQOL: Health-related quality of life
ITT: Intention-to-treat
MISS-21: Medical Interview Satisfaction Scale
PGIC: Patient Global Impression of Change
PQC-VAS: Perceived Quality of Communication
PRO: Patient reported outcomes
PROMs: Patient-reported outcome measures
QoL: Quality of life
RCT: Randomized controlled trial
SEIQoL: The Schedule for the Evaluation of Individual Quality of Life
SEIQoL-DW: The Schedule for the Evaluation of Individual Quality of Life-Direct Weighting
SF-36: Short Form-36
SIP: Sickness Impact Profile

## References

[1] Thorne S, Oliffe JL, Stajduhar KI (2013). Communicating shared decision-making: Cancer patient perspectives. Patient Education and Counseling.

[2] Street RL (2013). How clinician-patient communication contributes to health improvement: Modeling pathways from talk to outcome. Patient Education and Counseling.

[3] Meggiolaro E, Berardi MA, Andritsch E, Nanni MG, Sirgo A, Samori E, Farkas C, Ruffilli F, Caruso R, Belle M, Juan Linares E, de Padova S, Grassi L (2016). Cancer patients’ emotional distress, coping styles and perception of doctor-patient interaction in European cancer settings. Palliative & Supportive Care.

[4] Kunneman M, Engelhardt EG, Ten Hove FL, Marijnen CA, Portielje JE, Smets EM, de Haes HJ, Stiggelbout AM, Pieterse AH (2016). Deciding about (neo-)adjuvant rectal and breast cancer treatment: Missed opportunities for shared decision making. Acta Oncologica.

[5] Fallowfield L, Ratcliffe D, Jenkins V, Saul J (2001). Psychiatric morbidity and its recognition by doctors in patients with cancer. British Journal of Cancer.

[6] Detmar SB, Aaronson NK, Wever LD, Muller M, Schornagel JH (2000). How are you feeling? Who wants to know? Patients’ and oncologists’ preferences for discussing health-related quality-of-life issues. J Clin Oncol.

[7] De Vries AM, de Roten Y, Meystre C, Passchier J, Despland JN, Stiefel F (2014). Clinician characteristics, communication, and patient outcome in oncology: A systematic review. Psychooncology.

[8] Santana M-J, Feeny D (2014). Framework to assess the effects of using patient-reported outcome measures in chronic care management. Quality of Life Research.

[9] Yang LY, Manhas DS, Howard AF, Olson RA (2018). Patient-reported outcome use in oncology: A systematic review of the impact on patient-clinician communication. Support Care Cancer.

[10] Chen, Ou L, Hollis SJ (2013). A systematic review of the impact of routine collection of patient reported outcome measures on patients, providers and health organisations in an oncologic setting. BMC Health Services Research.

[11] Howell D, Li M, Sutradhar R, Gu S, Iqbal J, O'Brien MA, Seow H, Dudgeon D, Atzema C, Earle CC, DeAngelis C, Sussman J, Barbera L (2020). Integration of patient-reported outcomes (PROs) for personalized symptom management in “real-world” oncology practices: A population-based cohort comparison study of impact on healthcare utilization. Supportive Care in Cancer.

[12] Detmar SB, Muller MJ, Schornagel JH, Wever LD, Aaronson NK (2002). Health-related quality-of-life assessments and patient-physician communication: A randomized controlled trial. JAMA.

[13] Velikova G, Booth L, Smith AB, Brown PM, Lynch P, Brown JM, Selby PJ (2004). Measuring quality of life in routine oncology practice improves communication and patient well-being: A randomized controlled trial. Journal of Clinical Oncology.

[14] Rosenbloom SK, Victorson DE, Hahn EA, Peterman AH, Cella D (2007). Assessment is not enough: A randomized controlled trial of the effects of HRQL assessment on quality of life and satisfaction in oncology clinical practice. Psychooncology.

[15] Gill TM, Feinstein AR (1994). A critical appraisal of the quality of quality-of-life measurements. JAMA.

[16] Joyce, C. R. B., McGee, H. M., & O’Boyle, C. A. (Eds.) (1999). *Individual quality of life: Approaches to conceptualisation and assessment, vol xii*. Harwood Academic Publishers.

[17] Greenhalgh J, Dalkin S, Gooding K, Gibbons E, Wright J, Meads D, Black N, Valderas J, Pawson R (2017). Functionality and feedback: A realist synthesis of the collation, interpretation and utilisation of patient-reported outcome measures data to improve patient care. Health Services and Delivery Research.

[18] Greenhalgh J, Meadows K (1999). The effectiveness of the use of patient-based measures of health in routine practice in improving the process and outcomes of patient care: A literature review. Journal of Evaluation in Clinical Practice.

[19] O'Boyle, C., Hofer, S., & Ring, L. (Eds.) (2005). *Individualized quality of life. Assessing the quality of life in clinical trials*, (2nd ed., ). Oxford University Press.

[20] Greenhalgh, J., Long, A. F., & Flynn, R. (2005). The use of patient reported outcome measures in routine clinical practice: Lack of impact or lack of theory? *Social Science & Medicine*, *60*(4), 833–843. 10.1016/j.socscimed.2004.06.022. 10.1016/j.socscimed.2004.06.022 15571900

[21] Moons P, Marquet K, Budts W, De Geest S (2004). Validity, reliability and responsiveness of the “schedule for the evaluation of individual quality of life – Direct weighting” (SEIQoL-DW) in congenital heart disease. Health and Quality of Life Outcomes.

[22] Neudert C, Wasner M, Borasio GD (2001). Patients’ assessment of quality of life instruments: A randomised study of SIP, SF-36 and SEIQoL-DW in patients with amyotrophic lateral sclerosis. Journal of the Neurological Sciences.

[23] Hickey AM, Bury G, O'Boyle CA, Bradley F, O'Kelly FD, Shannon W (1996). A new short form individual quality of life measure (SEIQoL-DW): Application in a cohort of individuals with HIV/AIDS. BMJ.

[24] Hamidou Z, Baumstarck K, Chinot O, Barlesi F, Salas S, Leroy T, Auquier P (2017). Domains of quality of life freely expressed by cancer patients and their caregivers: Contribution of the SEIQoL. Health and Quality of Life Outcomes.

[25] Sprangers MA, Schwartz CE (1999). Integrating response shift into health-related quality of life research: A theoretical model. Social Science & Medicine.

[26] Aburub AS, Gagnon B, Rodríguez AM, Mayo NE (2016). Using a personalized measure (Patient Generated Index (PGI)) to identify what matters to people with cancer. Supportive Care in Cancer.

[27] Aburub AS, Mayo NE (2017). A review of the application, feasibility, and the psychometric properties of the individualized measures in cancer. Quality of Life Research.

[28] Kettis-Lindblad A, Ring L, Widmark E, Bendtsen P, Glimelius B (2007). Patients’ and doctors’ views of using the schedule for individual quality of life in clinical practice. The Journal of Supportive Oncology.

[29] Ring L, Kettis Lindblad A, Bendtsen P, Viklund E, Jansson R, Glimelius B (2006). Feasibility and validity of a computer administered version of SEIQoL-DW. Quality of Life Research.

[30] Nolte S, Liegl G, Petersen MA, Aaronson NK, Costantini A, Fayers PM, Groenvold M, Holzner B, Johnson CD, Kemmler G, Tomaszewski KA, Waldmann A, Young TE, Rose M (2019). General population normative data for the EORTC QLQ-C30 health-related quality of life questionnaire based on 15,386 persons across 13 European countries, Canada and the Unites States. European Journal of Cancer.

[31] Derogar M, van der Schaaf M, Lagergren P (2012). Reference values for the EORTC QLQ-C30 quality of life questionnaire in a random sample of the Swedish population. Acta Oncologica.

[32] Aaronson NK, Ahmedzai S, Bergman B, Bullinger M, Cull A, Duez NJ, Filiberti A, Flechtner H, Fleishman SB, de Haes JC (1993). The European Organization for Research and Treatment of Cancer QLQ-C30: A quality-of-life instrument for use in international clinical trials in oncology. JNCI: Journal of the National Cancer Institute.

[33] Fayers P, Bottomley A (2002). Quality of life research within the EORTC—The EORTC QLQ-C30. European Journal of Cancer.

[34] Peterman AH, Fitchett G, Brady MJ, Hernandez L, Cella D (2002). Measuring spiritual well-being in people with cancer: The functional assessment of chronic illness therapy--spiritual well-being scale (FACIT-Sp). Annals of Behavioral Medicine.

[35] Brady MJ, Peterman AH, Fitchett G, Mo M, Cella D (1999). A case for including spirituality in quality of life measurement in oncology. Psycho-Oncol.

[36] Munoz AR, Salsman JM, Stein KD, Cella D (2015). Reference values of the functional assessment of chronic illness therapy-spiritual well-being: A report from the American Cancer Society’s studies of cancer survivors. Cancer.

[37] Spitzer WO, Dobson AJ, Hall J, Chesterman E, Levi J, Shepherd R, Battista RN, Catchlove BR (1981). Measuring the quality of life of cancer patients: A concise QL-index for use by physicians. Journal of Chronic Diseases.

[38] Sloan JA, Aaronson N, Cappelleri JC, Fairclough DL, Varricchio C (2002). Assessing the clinical significance of single items relative to summated scores. Mayo Clinic Proceedings.

[39] Meakin R, Weinman J (2002). The ‘Medical Interview Satisfaction Scale’ (MISS-21) adapted for British general practice. Family Practice.

[40] Dworkin RH, Turk DC, Farrar JT, Haythornthwaite JA, Jensen MP, Katz NP, Kerns RD, Stucki G, Allen RR, Bellamy N, Carr DB, Chandler J, Cowan P, Dionne R, Galer BS, Hertz S, Jadad AR, Kramer LD, Manning DC, Martin S, McCormick CG, McDermott MP, McGrath P, Quessy S, Rappaport BA, Robbins W, Robinson JP, Rothman M, Royal MA, Simon L, Stauffer JW, Stein W, Tollett J, Wernicke J, Witter J (2005). Core outcome measures for chronic pain clinical trials: IMMPACT recommendations. Pain.

[41] Geisser ME, Clauw DJ, Strand V, Gendreau RM, Palmer R, Williams DA (2010). Contributions of change in clinical status parameters to Patient Global Impression of Change (PGIC) scores among persons with fibromyalgia treated with milnacipran. Pain.

[42] Mesa R, Verstovsek S, Kiladjian JJ, Griesshammer M, Masszi T, Durrant S, Passamonti F, Harrison CN, Pane F, Zachee P, Zhen H, Jones MM, Parasuraman S, Li J, Côté I, Habr D, Vannucchi AM (2016). Changes in quality of life and disease-related symptoms in patients with polycythemia vera receiving ruxolitinib or standard therapy. European Journal of Haematology.

[43] Yost KJ, Eton DT (2005). Combining distribution- and anchor-based approaches to determine minimally important differences: The FACIT experience. Evaluation & the Health Professions.

[44] Howell D, Molloy S, Wilkinson K, Green E, Orchard K, Wang K, Liberty J (2015). Patient-reported outcomes in routine cancer clinical practice: A scoping review of use, impact on health outcomes, and implementation factors. Annals of Oncology.

[45] Levati S, Campbell P, Frost R, Dougall N, Wells M, Donaldson C, Hagen S (2016). Optimisation of complex health interventions prior to a randomised controlled trial: A scoping review of strategies used. Pilot and Feasibility Studies.

[46] Santana MJ, Haverman L, Absolom K, Takeuchi E, Feeny D, Grootenhuis M, Velikova G (2015). Training clinicians in how to use patient-reported outcome measures in routine clinical practice. Quality of Life Research.

[47] Fagerlind H, Kettis A, Glimelius B, Ring L (2013). Barriers against psychosocial communication: oncologists’ perceptions. Journal of Clinical Oncology.

[48] Chong PF, Golledge J, Greenhalgh RM, Davies AH (2000). Exercise therapy or angioplasty? A summation analysis. European Journal of Vascular & Endovascular Surgery.

[49] Brundage MD, Wu AW, Rivera YM, Snyder C (2020). Promoting effective use of patient-reported outcomes in clinical practice: Themes from a “methods tool kit” paper series. Journal of Clinical Epidemiology.

